# A New Norisoprenoid and Other Compounds from Fuzhuan Brick Tea

**DOI:** 10.3390/molecules17033539

**Published:** 2012-03-19

**Authors:** Zhen-Mei Luo, Tie-Jun Ling, Li-Xiang Li, Zheng-Zhu Zhang, Hong-Tao Zhu, Ying-Jun Zhang, Xiao-Chun Wan

**Affiliations:** 1 Key Laboratory of Tea Biochemical and Biotechnology of Ministry of Education and Ministry of Agriculture, Anhui Agricultural University, Hefei 230036, China; Email: yeziluozhenmei@126.com (Z.-M.L.); ling_tiejun@yahoo.com.cn (T.-J.L.); 2 State Key Laboratory of Phytochemistry and Plant Resources in West China, Kunming Institute of Botany, Chinese Academy of Sciences, Kunming 650204, China

**Keywords:** Fuzhuan brick tea, norisoprenoid, 3*R*,9*R*-oxido-5-megastigmene, antimicrobial activity, enteric pathogenic microorganisms

## Abstract

Fuzhuan brick tea, a kind of dark tea consumed mainly in the border regions of Southwestern and Northwestern China since the 1860s, is produced from the leaves of *Camellia sinensis* var. *sinensis* by microbial fermentation. From this special fermented tea, a new norisoprenoid, 3*R*,9*R*-oxido-5-megastigmene, was isolated, together with *α*-linolenic acid, strictin, isovitexin, astragalin, (+)-catechin, (−)-epicatechin, (−)-epicatechin gallate, (+)-gallocatechin, (−)-epigallocatechin, (−)-epigallocatechin gallate and gallic acid. The structures of the compounds were identified by spectroscopic means. The new compound didn’t show any inhibition activity against the tested enteric pathogenic microorganisms at a concentration of 800 μg/mL by the hole plate diffusion method.

## 1. Introduction

Chinese commercial teas are classified into six categories: *i.e.*, green tea, oolong tea, black tea, white tea, yellow tea and dark tea, according to the different manufacturing process. Among them, dark tea is the only one that involves microbial fermentation in its manufacturing process [1]. Fuzhuan brick tea is one of the major brands of dark tea. It is mainly produced in Hunan province of China, and has been consumed by ethnic groups in the border regions of Southwestern and Northwestern China since the 1860s.

Fuzhuan brick tea is produced from the leaves of *Camellia sinensis* var. *sinensis*. The entire manufacturing process of the tea has been described in detail by Mo *et al.* and Xu *et al.* [2,3]. The fresh tea leaves are first pretreated to be raw dark green tea, which was then softened with steam and pressed into brick shapes before being placed in a fungal fermentation workshop for about 15-17 days. The “fungal fermentation” stage is unique to the manufacturing process of Fuzhuan brick tea. In the fermentation stage, many fungi grow within the tea leaves under controlled temperature and moisture conditions. The fungi growing during this stage were characterized as a mixture of several microorganisms with *Eurotium* spp., *Debaryomyces* spp. and *Aspergillus* spp. predominating [3]. Fungal growth is the key stage responsible for the unique functions of Fuzhuan brick tea, including anti-dysentery, anti-hyperlipidemia and anti-hyperglycemia [4,5,6]. Furthermore, it is interesting that the anti-dysentery activity of the tea is increased with the course of the fermentation [2,7]. Therefore, the bioactive compounds in the tea were implied to be formed by fermentation of these microbes [3,6,8]. Some rare 6-hydroxy-7-one oleanolic triterpenoids, and a epicatechin derivative with a 3,6-dihydro-6-oxo-2*H*-pyran-2-carboxylic acid moiety, were previously reported from Fuzhuan brick tea [9,10]. In our continuing research for the bioactive compounds from Fuzhuan brick tea, a new norisoprenoid was discovered, along with other 11 known compounds. We report herein the isolation and structural elucidation of these compounds.

## 2. Results and Discussion

**Figure 1:**
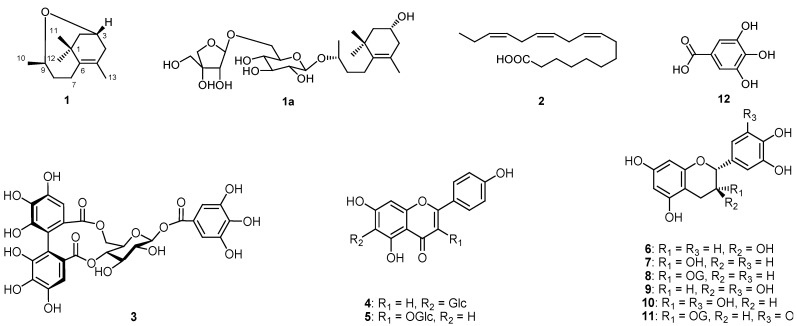
Structures of **1**–**12** and **1a**.

The 70% aqueous acetone extract of Fuzhuan brick tea was fractionated successively by partitioning with petroleum ether, CHCl_3_ and *n*-BuOH. The CHCl_3_ and *n*-BuOH fractions were separated by silica gel, Sephadex LH-20, ODS, polyamide and MCI-gel CHP20P column chromatrography (CC) to afford a new norisoprenoid (**1**) and 11 other known compounds, which were identified as *α*-linolenic acid (**2**) [11], strictin (**3**) [12,13], isovitexin (**4**) [14], astragalin (**5**) [15], (+)-catechin (**6**) [16], (−)-epicatechin (**7**) [16], (−)-epicatechin gallate (**8**) [16], (+)-gallocatechin (**9**) [16], (−)-epigallocatechin (**10**) [16], (−)-epigallocatechin gallate (**11**) [16], and gallic acid (**12**) (Figure 1), respectively, on the basis of their spectroscopic data and by comparison with the reference values and by authentic samples.

**Figure 2:**
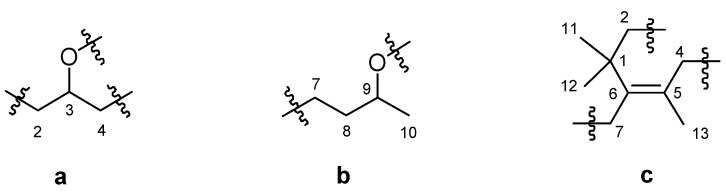
Structural fragments **a**–**c** of compound **1**.

**Figure 3:**
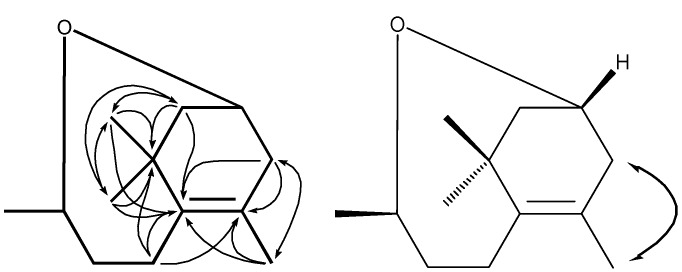
Key HMBC (^1^H→^13^C) and ROESY (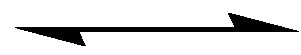) correlations of compound **1**.

Compound **1** was obtained as a colorless gum. The molecular formula of **1** was determined as C_13_H_22_O by the HRTOF-MS signal at *m/z* 194.1659 (calcd. 194.1671). The ^1^H-NMR of **1** showed two proton signals of oxygen bearing methine groups at δ 5.144 (1H, m) and 4.947 (1H, m), as well as the signals of one vinyl methyl at δ 1.490 (3H, s), one doublet methyl at 1.518 (3H, d, *J* = 4.8 Hz), and two quaternary methyls at 0.974 (6H, s). Its ^13^C-NMR and DEPT spectral data of 137.55 (s) and 124.58 (s) indicated the presence of olefinic group, which was supported by the IR spectral signal of 1,634 cm^−1^. The remaining 11 carbon signals were assigned by DEPT and HSQC as four methyls, four methylenes, two oxygen bearing methines, and one quaternary carbon. The ^1^H-^1^H COSY spectrum of **1** showed cross peaks between δ 2.26 (H-2a), 1.675 (H-2b) and δ 5.144 (H-3). The latter proton signal also showed COSY correlations with δ 2.727 (H-4a) and 2.337 (H-4b). These spectral features indicated a structural fragment of **a** (Figure 2). Similarly, another fragment **b** (Figure 2) could be determined by analysis of the COSY correlations between δ 2.337 (H-7a), 2.182 (H-7b), 1.861 (H-8a), 1.675 (H-8b), 4.947 (H-9), and 1.518 (H_3_-10). The HMBC spectrum of **1** showed correlations from δ 2.260 (H-2a), 1.675 (H-2b) and 0.974 (H_3_-11 and 12) to δ 37.97 (C-1) and 137.55 (C-6), as well as from δ 2.337 (H-7a), 2.182 (H-7b) to δ 37.97 (C-1), 124.58 (C-5) and 137.55 (C-6) (Figure 3). On the other hand, the HMBC correlations between the two olefinic carbon signals and δ 2.727 (H-4a) and 2.337 (H-4b), as well as δ 1.490 (H_3_-13) were observed. A further analysis of the ROESY correlation between H_2_-4 and H_3_-13 allowed the establishment of structural fragment **c** (Figure 2 and Figure 3). By combined analysis of the above evidences, **1** was indicated as a derivative of 3-hydroxy-7,8-dihydro-*β*-ionol [17,18,19]. The downfield shifts of ^13^C-NMR signals for C-3 and C-9 to δ 72.14 and 75.31 respectively, suggested an ether linkage between C-3 and C-9. There was no valuable information for stereochemistry of C-3 and C-9 in the ROESY spectrum. The stereochemistry of C-3 and C-9 were tentatively assigned to be *R* by comparison of the ^13^C-NMR data with those of the 3*R* or 9*R*-*O*-glycosylated 3-hydroxy-7,8-dihydro-*β*-ionol derivatives [20,21,22]. This was also supported by the presence of (3*R*,9*R*)-3-hydroxy-7,8-dihydro-*β*-ionyl 6-*O*-*β*-D-apiofuranosyl-*β*-D-glucopyranoside (**1a**), which was supposed to be the precursor of **1** in fresh tea leaves [19]. On the basis of these evidences, **1** was identified as 3*R*,9*R*-oxido-5-megastigmene.

Compound **1a** was reported as an aroma precursor from the fresh tea leaves [19], while compound **1** was the C-3/C-9 ether derivative of **1a** after the sugar moiety was removed. It is interesting that **1** may be formed by microbial fermentation of Fuzhuan brick tea with dehydration of 3-OH and 9-OH on 3-hydroxy-7,8-dihydro-*β*-ionol, which is liberated from its precursor (**1a**) by endogenous enzymes during the manufacturing process of fresh tea leaves [19].

Since Fuzhuan brick tea is produced by microbe fermentation, the isolated new compound **1** was evaluated for its antibacterial activity against four enteric pathogenic microorganisms, *i.e.*, enteropathogenic *Escherichia coli* (EPEC), *Staphyloccocus aureus*, *Shigella dysenteriae*, and *Salmonella typhi*, by the hole plate diffusion method [9]. However, the compound did not exhibit any activity against the above microorganisms.

## 3. Experimental

### 3.1. General

Optical rotation was measured on a P-1020 Polarimeter (Jasco, Tokyo, Japan). IR spectra were measured on an IR-450 spectrometer (Shimadzu, Kyoto, Japan). ^1^H- and ^13^C-NMR, ^1^H-^1^H COSY, HSQC, HMBC and ROESY spectra were recorded with DRX-500 and Bruker AM-400 spectrometers operating at 500 and 400 MHz for ^1^H, and 125 and 100 MHz for ^13^C, respectively. Coupling constants were expressed in Hz. The high resolution time-of-flight-mass spectrometry (HRTOF-MS) was recorded on a GCT-MS instrument (Micromass Ltd., Manchester, UK) by direct inlet. Silica gel 60 (200–300 mesh, Qingdao Marine Chemical CO. Ltd., Qingdao, China), Sephadex LH-20 (25–100 μm, Pharmacia Fine Chemical Co., Ltd., Uppsala, Sweden), YMC GEL ODS-A (50 μm, YMC Co. Ltd., Kyoto, Japan), polyamide (100–200 mesh, Luqiaosijia Biochemical Co., Ltd., Zhejiang, China), and MCI-gel CHP20P (20–100 μm, Mitsubishi Chemical Co., Ltd., Tokyo, Japan) were used for CC. Aqueous MeOH from 0 to 100% (v/v) in increments of 10% was used as eluant for all Sephadex LH-20 and MCI-gel CC. TLC was performed on precoated kieselgel 60 F254 plates (Merck, Darmstadt, Germany), with chloroform/methanol/H_2_O (14:3:0.5, v/v), or benzene/ethyl formate/formoic acid (3:6:1 or 2:7:1, v/v), and detected by spraying with 2% ethanolic FeCl_3_ or 5% ethanolic H_2_SO_4_ reagent followed by heating.

### 3.2. Materials

Fuzhuan brick tea (produced in December, 2006) was purchased from Yiyang Fu Cha Industry Development Co. Ltd. (Yiyang, Hunan Province, China). Ampicillin sodium was purchased from Sigma Chemical Co. Ltd. (St. Louis, Missouri, USA). The test microorganisms were obtained from School of Basic Medical Sciences, Anhui Medical University (Hefei, China).

### 3.3. Extraction and Isolation

Fuzhuan brick tea (3.6 kg) was processed as described before [9] to obtain petroleum ether and chloroform fractions. The remaining aqueous solution was fractionated with *n*-BuOH. The chloroform solution was concentrated to afford a dark green residue (460 g), which was separated by silica gel and ODS CC to give **2** (55 mg). The *n*-BuOH solution was concentrated under reduced pressure to afford a brown residue (500 g). This residue was applied to a Sephadex LH-20 CC, yielding three subfractions (S1-S3). S1 was separated by combination of MCI-gel and ODS CC to give **1** (12 mg). S2 was subjected to a Sephadex LH-20 CC and a polyamide CC to yield **4** (30 mg) and **5**(25 mg). S3 was separated by repeated Sephadex LH-20 and MCI-gel CC, yielding **3** (15 mg), **6** (110 mg), **7** (120 mg), **8** (50 mg), **9** (85 mg), **10** (95 mg), **11** (100 mg), and **12** (3 g).

### 3.4. 3,9-Oxido-5-megastigmene

Colorless gum, [*α*]−3.6 (*c* 0.09, MeOH). IR (KBr) *ν*_max_ (cm^−1^): 2959, 2925, 1634, 1465, 1379, 1339, 1280, 1174, 1144, 611. EI-MS *m/z* (%): 194 (3.9), 176 (12.4), 161 (21.6), 121 (45.6), 119 (100.0), 105 (52.5), 91 (51.8), 79 (24.3), 65 (11.6), 55 (47.2), 41 (25.4). HRTOF-MS: *m/z* 194.1659 (M^+^, calcd. for C_13_H_22_O^+^, 194.1671). ^1^H- and ^13^C-NMR data, see Table 1.

**Table 1:** NMR data of 1 (in CDCl_3_, δ in ppm, J in Hz).

Positions	δ_H_	δ_C_	HMBC (^1^H to ^13^C)
1	─	37.97	
2	2.260 (1H, br d, 9.6, H-2a)	46.37	C-1, 3, 4, 6, 11, 12
	1.675 (1H, m, H-2b) *^a ^*		C-1, 3, 4, 6, 11, 12
3	5.144 (1H, m)	72.14	
4	2.727 (1H, dd, 12.8, 3.6, H-4a)	40.44	C-2, 3, 5, 6, 13
	2.337 (1H, m, H-4b) *^b^*		C-2, 3, 5, 6, 13
5	─	124.58	
6	─	137.55	
7	2.337 (1H, m, H-7a) *^b^*	24.56	C-1, 5, 6, 8, 9
	2.182 (1H, ddd, 10.4, 10.4, 3.6, H-7b)		C-1, 5, 6, 8, 9
8	1.861 (1H, m, H-8a)	38.22	C-7, 9, 10
	1.675 (1H, m, H-8b) *^a^*		C-7, 9, 10
9	4.947 (1H, m)	75.31	C-7, 8
10	1.518 (3H, d, 4.8)	21.39	C-8, 9
11	0.974 (3H, s) *^c^*	28.53 *^d^*	C-1, 2, 6, 12
12	0.974 (3H, s) *^c^*	29.81 *^d^*	C-1, 2, 6, 11
13	1.490 (3H, s)	19.84	C-4, 5, 6

*^a b c^* Signals were overlapped; *^d^* Signals may be interchanged.

### 3.5. Antibacterial Assays

The antibacterial activity of **1** against four enteric pathogenic microorganisms, *i.e.*, enteropathogenic *E . coli* (EPEC), *S. aureus*, *S. dysenteriae*, and *S. typhi* were evaluated by the hole plate diffusion method as described before [9]. Compound **1** and ampicillin sodium (positive control) were individually dissolved and diluted with DMSO to obtain serial concentrations of 800, 400, 200, 100, 50, 25 μg/mL. DMSO was used as the negative control. Three 6 mm wide holes were bored with a sterilized steel borer into the Nutrient Agar Media (beef extract 3 g, peptone 10 g, agar 17 g, NaCl 5 g, H_2_O 1,000 mL, pH 7.2) in each Petri dish inoculated with the test microorganism. The solution of the compound (60 μL) at a specific concentration was added into each of the holes. DMSO was used as the negative control. The plates were then incubated at 37 °C for 24 h. The inhibition zones around the holes were measured and the minimal inhibitory concentration (MIC), which was defined as the lowest concentration able to inhibit any visible bacterial growth, was recorded. The assays were performed three times in order to guarantee reproducibility of results. Compound **1** did not exhibit any activity against the four tested microorganisms at a concentration of 800 μg/mL.

## 4. Conclusions

Fungal aroma, which is typical of Fuzhuan brick tea, is produced by a fungal growth stage in the production process [23]. Given the different odors of norisoprenoids and their ethers, the generation and occurrence of compound **1** may partially contribute to the unique aroma property of the tea [24,25,26]. Fuzhuan brick tea has remarkable anti-dysentery activity, which is increased with the course of the microbial fermentation [2,7]. Since the amount of polyphenolics decreased during fermentation, there should be some non-phenolic anti-microbial compounds produced by the dominant fungi in this tea [27]. Though some compounds with interesting structures have been discovered by now, the unique bio-functions of the tea have not been explained yet. Therefore, more phytochemical and bioassay works need to be performed on Fuzhuan brick tea.

## Supplementary Materials

Supplementary materials can be accessed at: http://www.mdpi.com/1420-3049/17/3/3539/s1.
