# Supplementary material for: A New Norisoprenoid and Other Compounds from Fuzhuan Brick Tea
Source: Molecules. 2012 Mar 19;17(3):3539–46. doi: 10.3390/molecules17033539 (PMC6268391; doi:10.3390/molecules17033539)
Supplement: Supplementary file 1 [file molecules-17-03539-s001.pdf]

Supplementary Materials

Article

A New Norisoprenoid and Other Compounds from Fuzhuan Brick Tea

Zhen-Mei Luo [1,†], Tie-Jun Ling [1,†], Li-Xiang Li [1], Zheng-Zhu Zhang [1], Hong-Tao Zhu [2], Ying-Jun Zhang [2,*] and Xiao-Chun Wan [1,*]

1 Key Laboratory of Tea Biochemical and Biotechnology of Ministry of Education and Ministry of Agriculture, Anhui Agricultural University, Hefei 230036, China; E-Mails: yeziluozhenmei@126.com (Z.-M.L.); ling_tiejun@yahoo.com.cn (T.-J.L.)

2 State Key Laboratory of Phytochemistry and Plant Resources in West China, Kunming Institute of Botany, Chinese Academy of Sciences, Kunming 650204, China

† These authors contributed equally to the work.

* Authors to whom correspondence should be addressed: E-Mails: zhangyj@mail.kib.ac.cn (Y.-J.Z.); xcwan@ahau.edu.cn (X.-C.W.); Tel.: +86-871-522-3235 (Y.-J.Z.); Fax: +86-871-515-0124 (Y.-J.Z.); Tel./Fax: +86-551-578-6765 (X.-C.W.).

The supplementary materials for the manuscript:
A new norisoprenoid derivative and other compounds from Fuzhuan brick tea

^{1}H NMR spectrum:

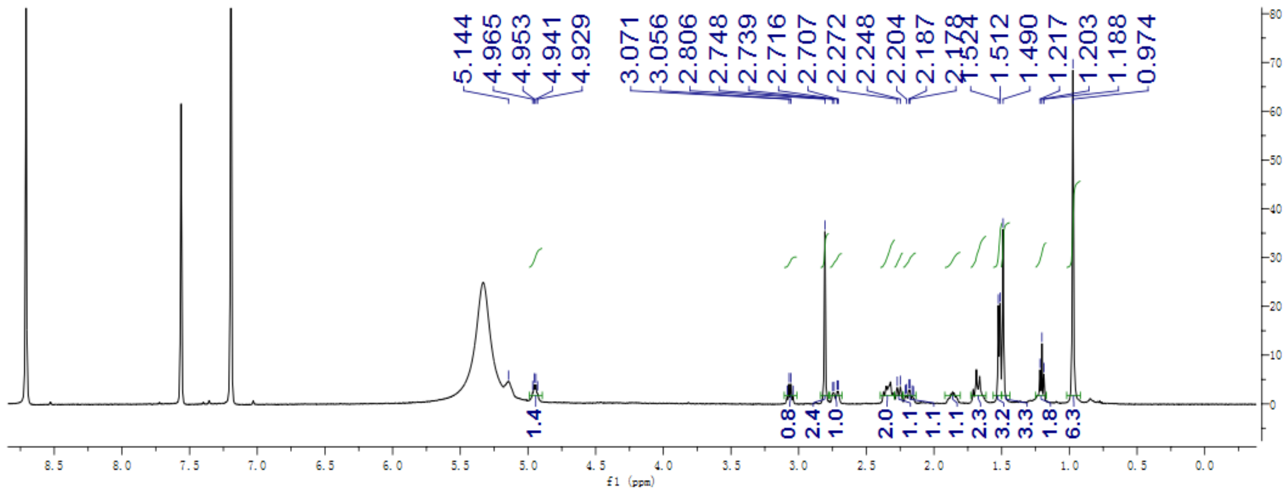

^{13}C NMR spectrum

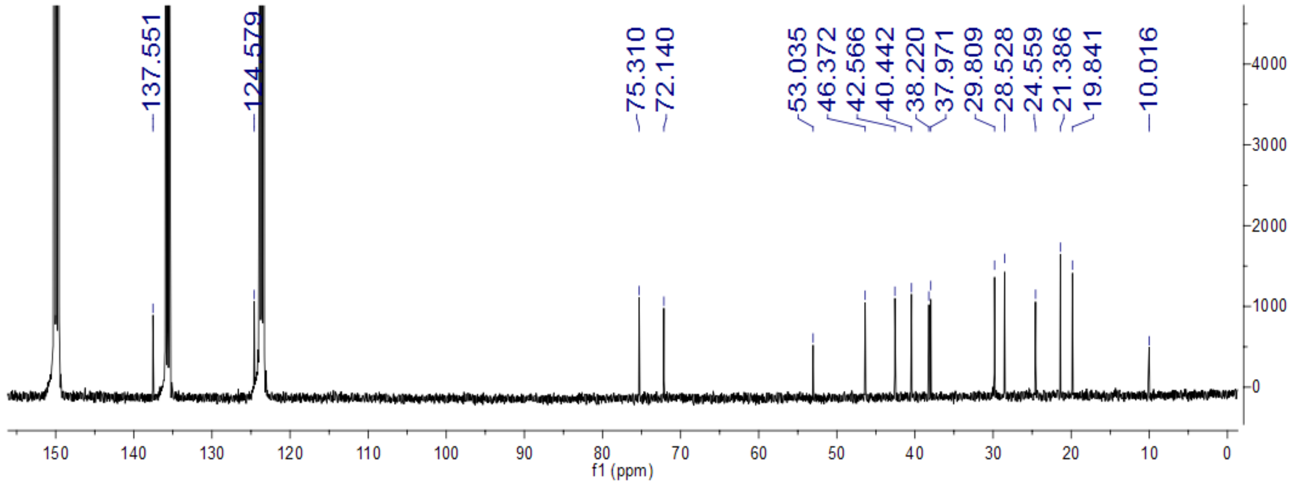

DEPT 135

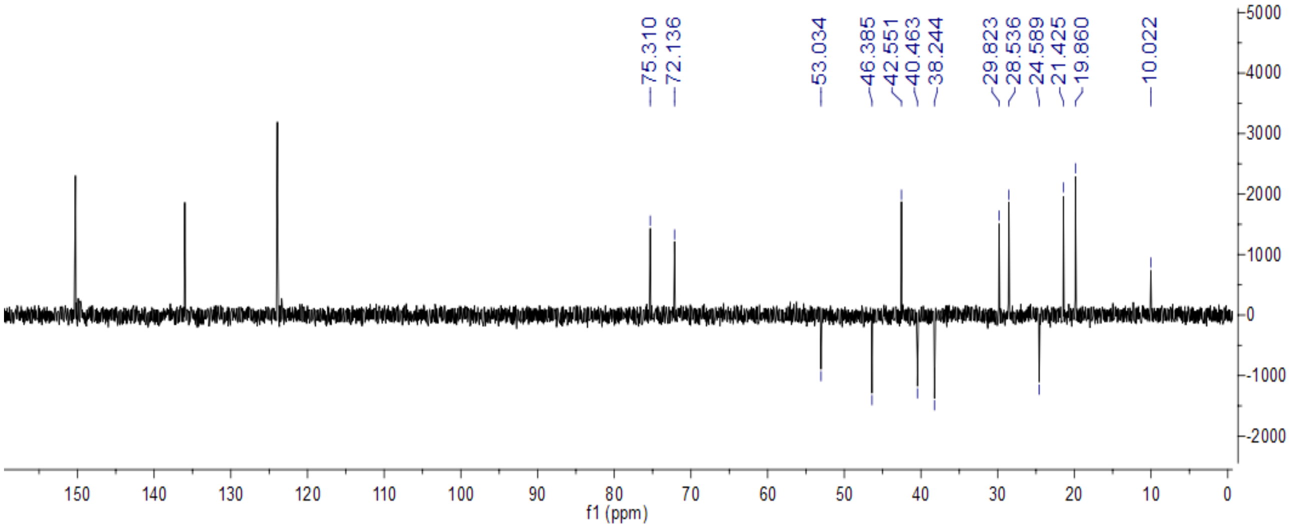

DEPT 90

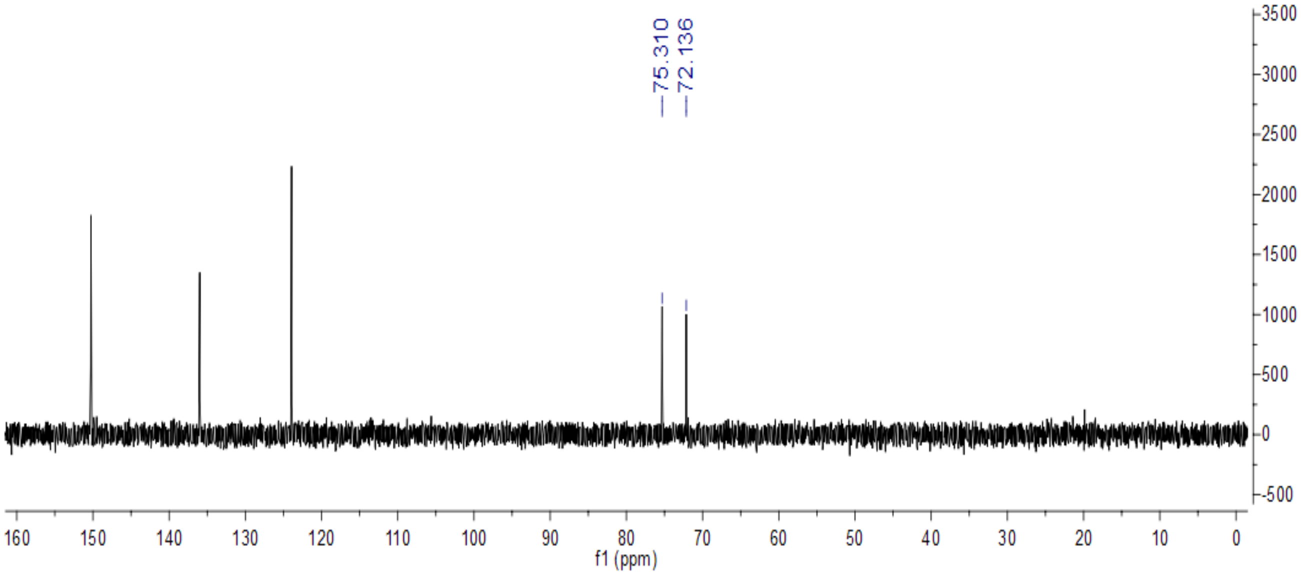

ROESY

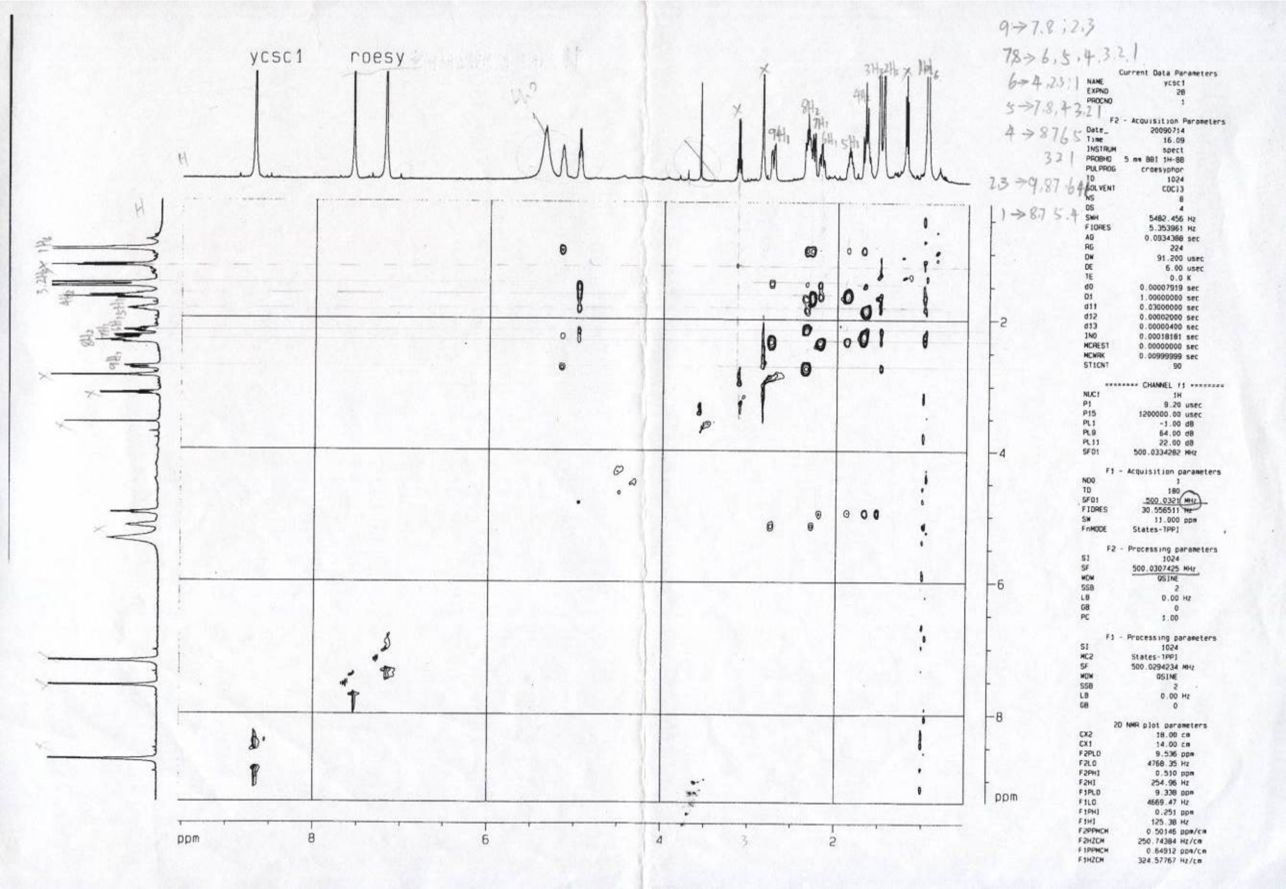

^{1}H-^{1}H COSY

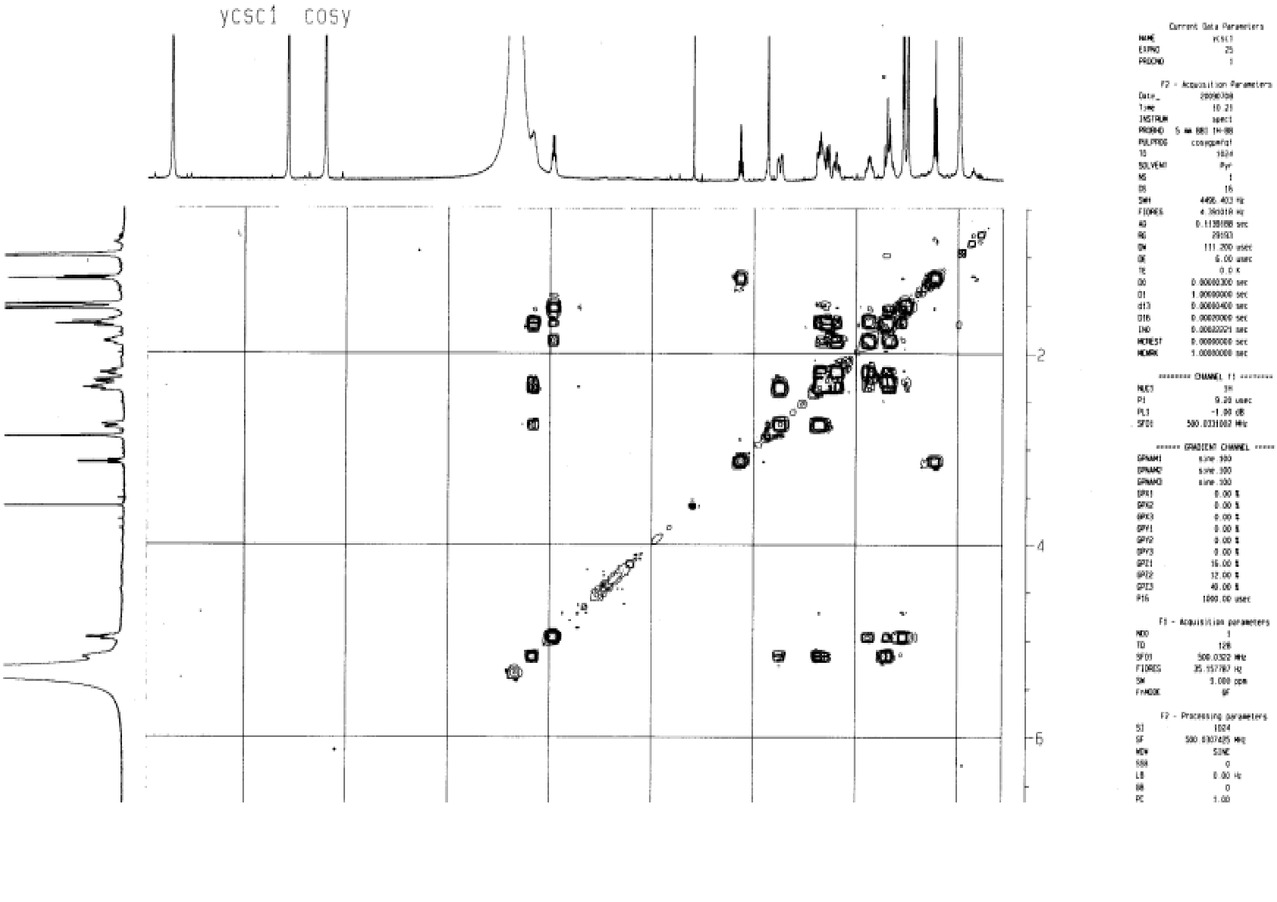

HMBC

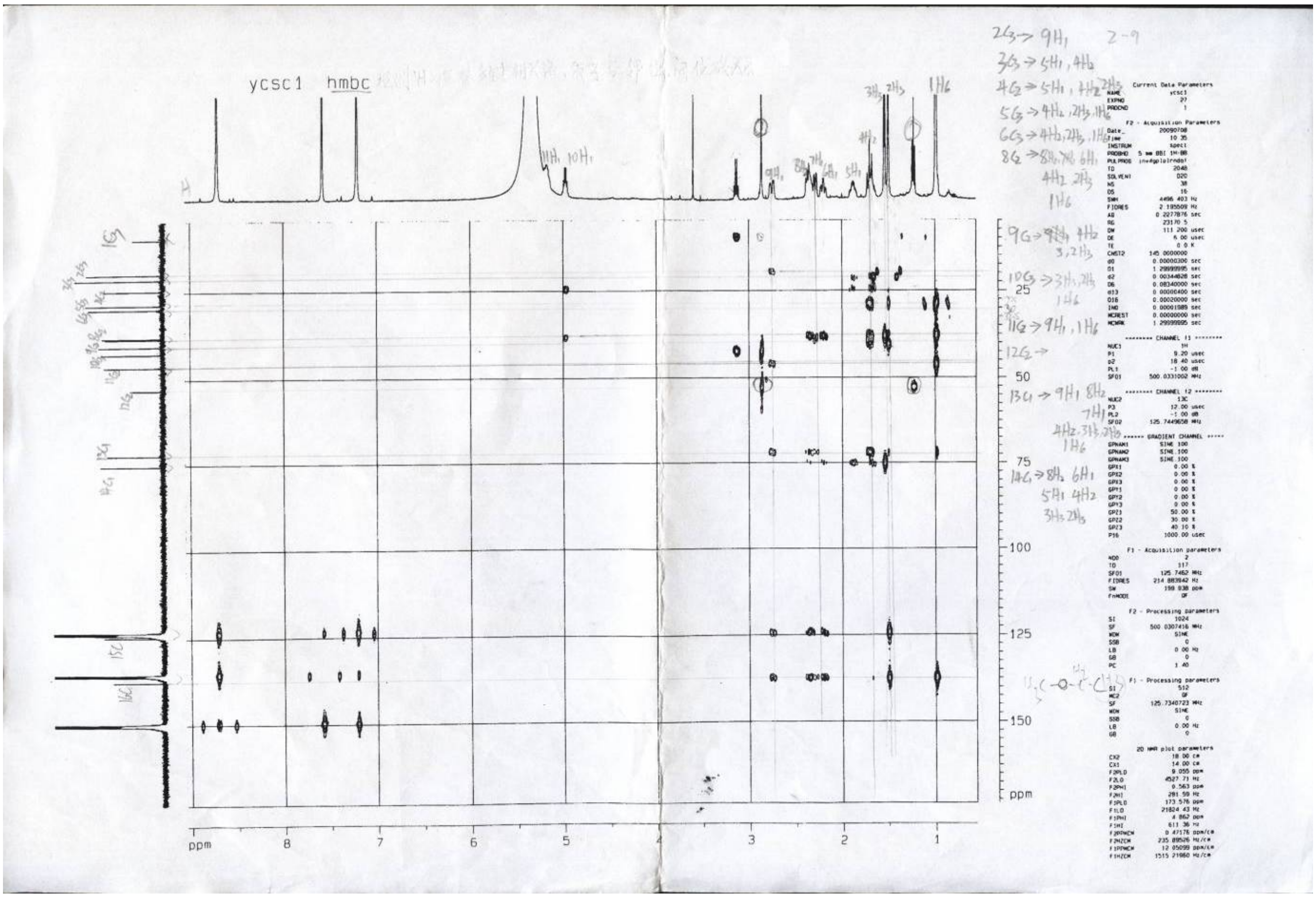

HSQC

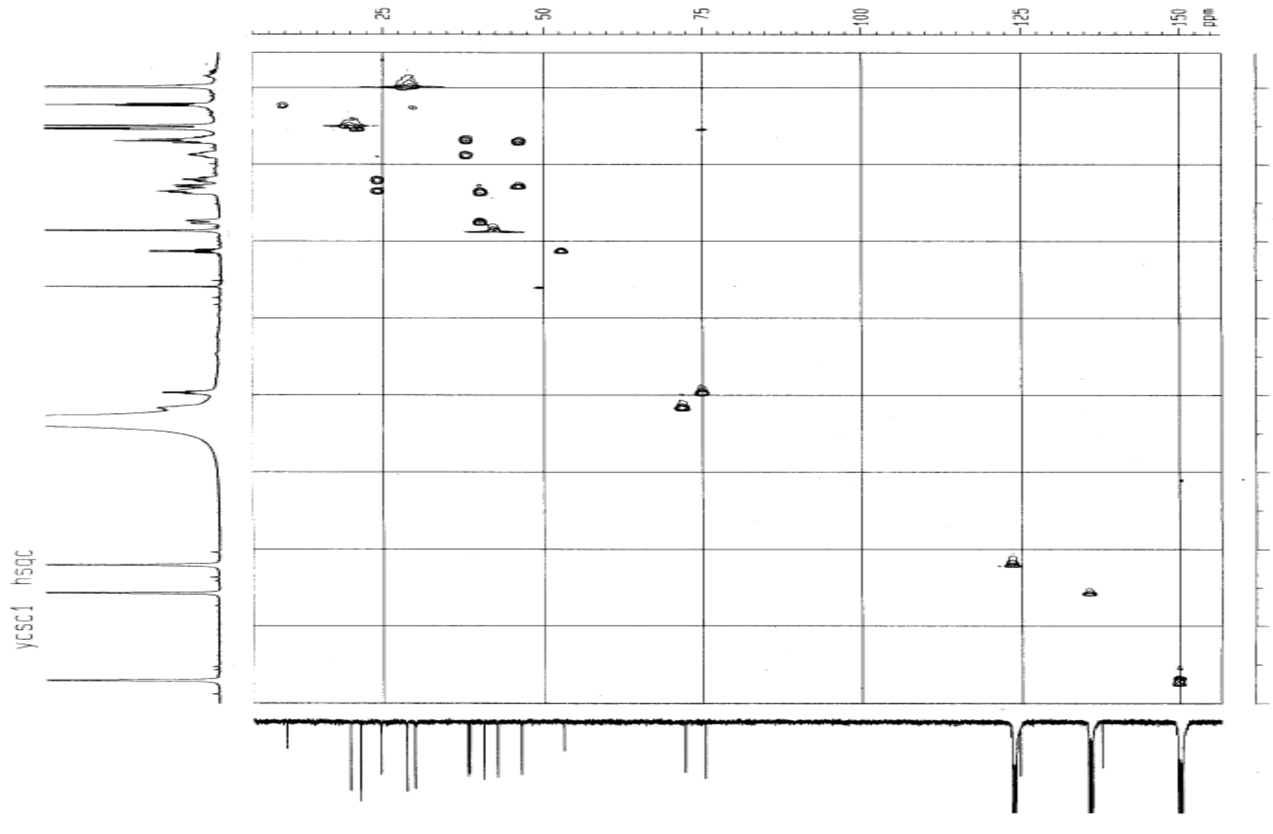

HRTOFMS

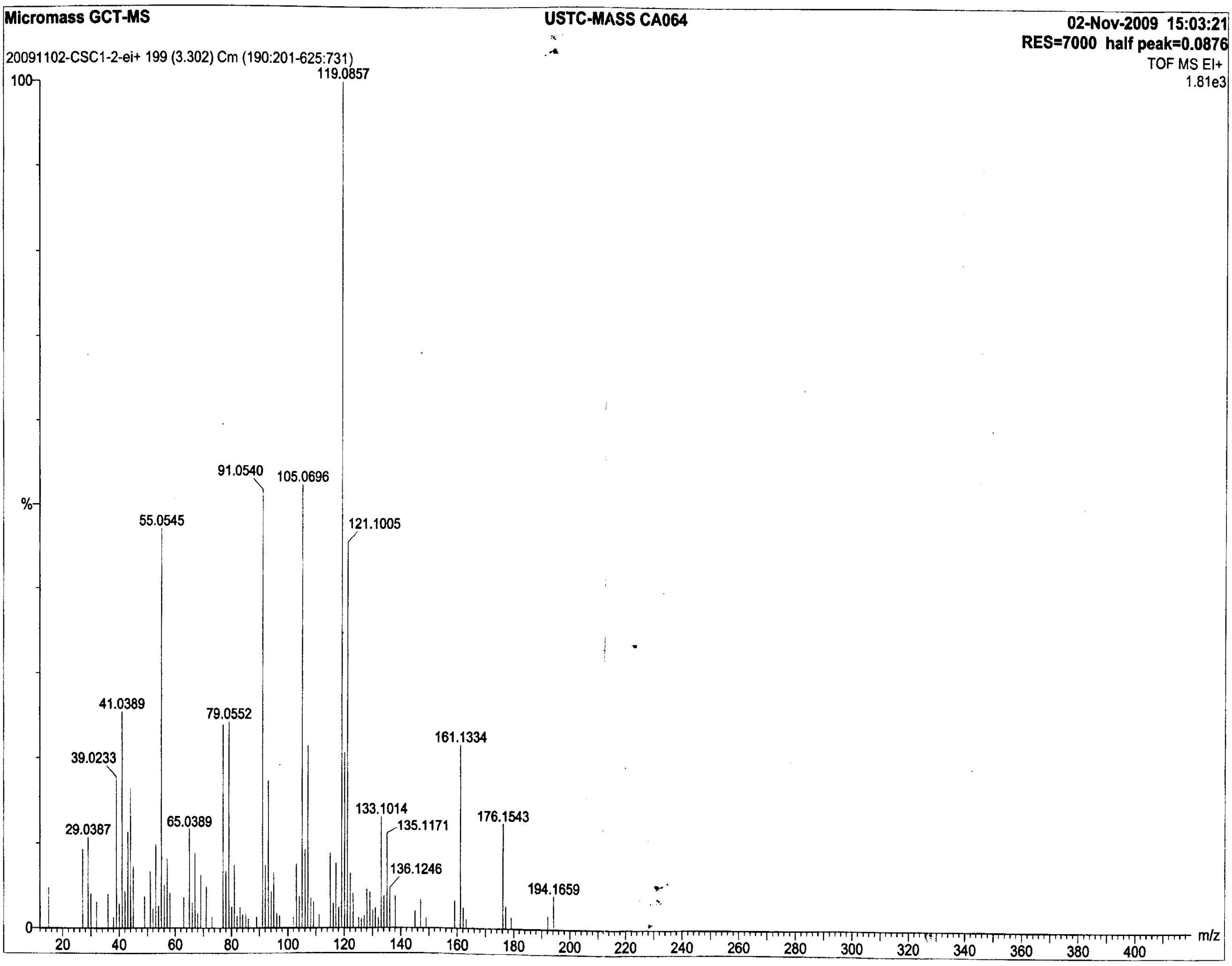

Elemental Composition Report **Page 2**

Multiple Mass Analysis: 96 mass(es) processed - displaying only valid results
Tolerance = 8.0 mDa / DBE: min = -1.0, max = 50.0

Monoisotopic Mass, Odd and Even Electron Ions
503 formula(e) evaluated with 85 results within limits (up to 50 closest results for each mass)

Minimum:	1.00				-1.0	
Maximum:	100.00		8.0	60.0	50.0	
Mass	RA	Calc. Mass	mDa	PPM	DBE	Formula
131.0885	2.36	131.0861	2.4	18.5	5.5	C10 H11
132.0935	1.12	132.0939	-0.4	-3.0	5.0	C10 H12
133.1014	13.25	133.1017	-0.3	-2.4	4.5	C10 H13
134.1101	3.75	134.1096	0.5	4.1	4.0	C10 H14
135.1171	11.24	135.1174	-0.3	-2.0	3.5	C10 H15
136.1246	4.75	136.1252	-0.6	-4.4	3.0	C10 H16
138.1064	3.80	138.1045	1.9	14.0	3.0	C9 H14 O
145.0971	2.01	145.1017	-4.6	-31.9	5.5	C11 H13
147.1138	3.36	147.1174	-3.6	-24.3	4.5	C11 H15
159.1180	3.24	159.1174	0.6	3.9	5.5	C12 H15
161.1334	21.64	161.1330	0.4	2.3	4.5	C12 H17
162.1366	2.40	162.1409	-4.3	-26.2	4.0	C12 H18
163.1487	1.06	163.1487	0.0	0.1	3.5	C12 H19
176.1543	12.36	176.1565	-2.2	-12.5	4.0	C13 H20
177.1572	2.63	177.1643	-7.1	-40.2	3.5	C13 H21
179.1387	1.29	179.1436	-4.9	-27.3	3.5	C12 H19 O
192.1509	1.57	192.1514	-0.5	-2.7	4.0	C13 H20 O
194.1659	3.91	194.1671	-1.2	-6.0	3.0	C13 H22 O
